# Rapid clinical response to nemolizumab in a patient with lichen amyloidosis

**DOI:** 10.1016/j.jdcr.2026.05.021

**Published:** 2026-05-15

**Authors:** Kaito Iwama, Daiki Rokunohe, Kazuhito Kogawa, Eijiro Akasaka

**Affiliations:** Department of Dermatology, Hirosaki University Graduate School of Medicine, Hirosaki, Japan

**Keywords:** atopic dermatitis, interleukin 31, itch, lichen amyloidosis, nemolizumab, primary localized cutaneous amyloidosis

Lichen amyloidosis (LA) is characterized by pruritic, hyperkeratotic papules typically affecting the extensor surfaces of extremities and the interscapular region in middle-aged and elderly adults. We report a case of refractory LA that showed rapid and remarkable improvement with nemolizumab, a humanized monoclonal antibody against interleukin (IL) 31 receptor A.

## Case report

**Fig 1:**
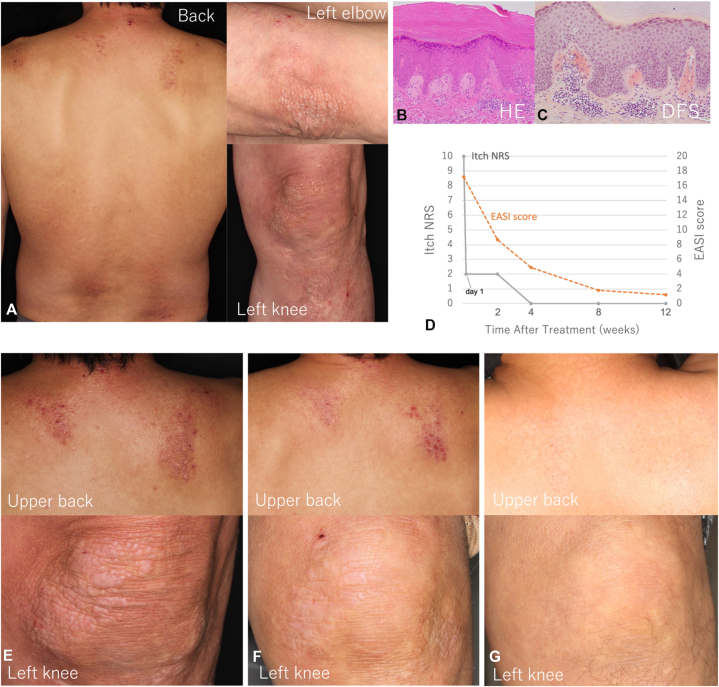
**A,** Solid papules were found on the elbows, knees, and upper back. **B, C**, Biopsy specimen from the left elbow revealed amorphous deposits in the dermal papillae and staining positively with direct fast scarlet. **D**, Decrease of itch numerical rating scale (NRS) and eczema area and severity index score after nemolizumab treatment. Clinical manifestations of, **E**, immediately before, **F**, 2 weeks after, and **G**, 12 weeks after the initiation of nemolizumab. (**B** and **C,** direct fast scarlet; original magnifications: **B,** '200; **C,** '200.)

A 60-year-old man was referred to our department for a severe itchy skin eruption, with an itch numerical rating scale (NRS) of 9/10. He also suffered from sleep disturbance due to the pruritus. He had been treated for over 10 years with a diagnosis of atopic dermatitis (AD), receiving topical corticosteroids (betamethasone butyrate propionate ointment) and oral antihistamines without significant improvement. Physical examination revealed multiple coalescing solid papules on both the elbows, knees, and upper back (Fig 1, *A*). The skin was generally rough and dry, with eczematous and lichenified lesions on the lower back and dorsal hands. Considering the clinical presentation and the intractable pruritus, the patient was diagnosed with LA.

Skin biopsy from the left elbow revealed amorphous deposits in the papillary dermis, which stained positive with direct fast scarlet, confirming amyloid deposition (Fig 1, *B, C*). We performed laboratory tests to evaluate the underlying AD and to assess the systemic Th2 inflammatory status of the patient, showing normal eosinophil count, thymus and activation-regulated chemokine, and total immunoglobulin E levels. No clinical findings suggesting systemic amyloidosis were observed. The patient was diagnosed with LA coexisting with AD features. The patient underwent 2 months of intensified topical therapy. This included comprehensive education on proper application techniques—ensuring the correct amount and frequency of topical corticosteroid application—as well as occlusive dressing therapy with heparinoid cream. The type of topical corticosteroid was not changed. The papules partially flattened; however, intense pruritus persisted (itch NRS 10/10), and the eczema area and severity index score was 17.2 (Fig 1, *E*). After discussing the limited evidence, we administered nemolizumab 60 mg subcutaneously every 4 weeks. Remarkably, pruritus improved from NRS 10/10 to 2/10 the following day (Fig 1, *D*). Two weeks after administration, the papules notably flattened (Fig 1, *F*). By 4 weeks, pruritus had completely resolved (itch NRS 0/10), and at 12 weeks, the eczema area and severity index score decreased to almost zero (Fig 1, *G*). There were no adverse effects from nemolizumab. Treatment was discontinued at 12 weeks because of financial constraints, but his LA and AD maintained improvement (itch NRS 0-1) for 12 weeks after treatment. Thereafter, pruritus recurred, and nemolizumab was restarted 17 weeks after discontinuation. Pruritus remained well controlled for approximately 1 year without any adverse events.

## Discussion

LA is a chronic condition in which repeated scratching stimulation leads to local inflammation. Inflammatory mediators released during this process further exacerbate the itch, resulting in persistent pruritus.^1^ Targeting the itch-scratch cycle is crucial for effective treatment; however, its management is often difficult. Previous reports have demonstrated increased expression of IL-31 in the cutaneous lesions of LA,^2^ as well as a case of hereditary skin amyloidosis caused by a point mutation in the IL-31 receptor A gene.^3^ These suggest that IL-31 may play an important role in the pathogenesis of primary localized cutaneous amyloidosis. To date, only a few case reports have been published on the use of the anti-IL-31 receptor A antibody, nemolizumab, for the treatment of primary localized cutaneous amyloidosis.^4^, ^5^, ^6^ In these reports, relatively rapid responses to nemolizumab were noted: itch was resolved between 5 days and 6 weeks after treatment initiation. The immediate response was also remarkable in our case, with the itch NRS decreasing from 10/10 to 2/10 within 1 day of initial administration. This rapid response contrasts with conventional therapies and suggests that direct blockade of the IL-31-mediated pathway was effective in inhibiting the itch-scratch cycle in our patient.

However, the pathogenesis of LA is complex, involving multiple chemical mediators. Dupilumab, which inhibits IL-4/IL-13-mediated signaling, has also demonstrated therapeutic benefits in LA, particularly in patients with atopic features.^7^^,^^8^ Indeed, one report noted that, in addition to IL-31, IL-13 was highly expressed in the lesional skin of a patient with LA.^2^ On the other hand, a previous report described a patient with nonatopic LA whose pruritus was relieved within 5 days after initiation of nemolizumab, despite being refractory to prior dupilumab treatment for 5 months.^5^ Although Soto-Canetti et al^5^ reported nemolizumab efficacy in a patients with nonatopic LA refractory to dupilumab, and Nakagawa et al^6^ described its use in LA, our case uniquely demonstrates a striking therapeutic response in a patient with coexisting LA and AD features despite normal serum IgE and thymus and activation-regulated chemokine levels at treatment initiation. This finding indicates that nemolizumab can be highly effective even when conventional biomarkers of atopic disease are unremarkable, suggesting that the dominant clinical phenotype—rather than laboratory atopic markers—should guide the decision to target the IL-31 pathway directly.

Furthermore, our case provides detailed longitudinal data on treatment discontinuation and retreatment, which has not been thoroughly addressed in prior reports.^4^, ^5^, ^6^ After achieving complete remission at 12 weeks, nemolizumab was discontinued. The patient remained symptom-free for an additional 12 weeks, suggesting that sustained interruption of the itch-scratch cycle may induce a period of prolonged remission even after cessation of therapy. However, pruritus eventually recurred 17 weeks after discontinuation, necessitating retreatment. Upon restarting nemolizumab, pruritus remained well controlled for approximately 1 year without adverse events. These observations suggest 2 important clinical implications: (1) short-term nemolizumab treatment can break the itch-scratch cycle and induce extended remission, and (2) continuous or intermittent maintenance therapy is ultimately necessary to achieve durable long-term remission in refractory LA.

Further investigation into optimal dosing intervals and predictors of sustained remission would be required to determine treatment strategies for refractory LA.

## Conflicts of interest

None disclosed.
